# Diagnostic method-based underestimation of leptospirosis in clinical and research settings; an experience from a large prospective study in a high endemic setting

**DOI:** 10.1371/journal.pntd.0010331

**Published:** 2022-04-04

**Authors:** Janith Warnasekara, Shalka Srimantha, Chamila Kappagoda, Dinesha Jayasundara, Indika Senevirathna, Michael Matthias, Suneth Agampodi, Joseph M. Vinetz

**Affiliations:** 1 Department of Community Medicine, Faculty of Medicine and Allied Sciences, Rajarata University of Sri Lanka, Saliyapura, Sri Lanka; 2 Department of Microbiology, Faculty of Medicine and Allied Sciences, Rajarata University of Sri Lanka, Saliyapura, Sri Lanka; 3 Department of Biochemistry, Faculty of Medicine and Allied Sciences, Rajarata University of Sri Lanka, Saliyapura, Sri Lanka; 4 Section of Infectious Diseases, Department of Internal Medicine, School of Medicine, Yale University, New Haven, Connecticut, United States of America; Lowell General Hospital, UNITED STATES

## Abstract

**Background:**

Leptospirosis has globally significant human mortality and morbidity, yet estimating the clinical and public health burden of leptospirosis is challenging because timely diagnosis remains limited. The goal of the present study was to evaluate leptospirosis undercounting by current standard methods in both clinical and epidemiological study settings.

**Methodology/Principal findings:**

A prospective hospital-based study was conducted in multiple hospitals in Sri Lanka from 2016 to 2019. Culture, whole blood, and urine samples were collected from clinically suspected leptospirosis cases and patients with undifferentiated fever. Analysis of biological samples from 1,734 subjects confirmed 591 (34.1%) cases as leptospirosis and 297 (17.1%) were classified as “probable” leptospirosis cases. Whole blood quantitative PCR (qPCR) did identify the most cases (322/540(60%)) but missed 40%. Cases missed by each method include; urine qPCR, 70% (153/220); acute sample microscopic agglutination test (MAT), 80% (409/510); paired serum sample MAT, 58% (98/170); and surveillance clinical case definition, 53% (265/496). qPCR of negative culture samples after six months of observation was of diagnostic value retrospectively with but missed 58% of positives (109/353).

**Conclusion:**

Leptospirosis disease burden estimates should consider the limitations of standard diagnostic tests. qPCR of multiple sample types should be used as a leading standard test for diagnosing acute leptospirosis.

## Author summary

Diagnostics of leptospirosis have not been optimised yet and is considered as a significant limiting factor for estimating the disease burden. This prospective hospital-based study, including 1734 clinically suspected leptospirosis cases and undifferentiated febrile patients, revealed that a minimum of 40% of cases would be missed by using any of the following tests individually. (Whole blood qPCR, single or paired-sample MAT, Urine qPCR, culture, culture qPCR, surveillance case definition). Therefore, we conclude that whole blood qPCR should be the standard test for diagnosing leptospirosis for clinical purposes until day 10 of the reported disease. MAT should be limited to places where the serological diagnosis has an epidemiological interest. qPCR testing of microscopically-negative cultures should be done before discarding to increase the yield in research settings.

## Introduction

Leptospirosis is a major zoonotic disease-causing significant mortality and morbidity, especially in tropical countries [1]. Globally, leptospirosis has been estimated to be responsible for 58,900 deaths and nearly one million cases annually [2]. However, analysis of the global disease burden of leptospirosis underestimates the actual toll because of limited standardised prospective surveillance and reporting to public health authorities [2, 3]. A major systematic review conducted to assess the disease burden highlighted that sparsely distributed, non-representative data, deficiencies of diagnostic tests, and non-inclusion of mild or asymptomatic cases are among the reasons for underestimating the public health impact of leptospirosis worldwide [2].

Pathogenic *Leptospira* spp. colonise reservoir host proximal convoluted renal tubules and are excreted into the environment through the urine [4]. Once *Leptospira* are shed to the environment, they may enter the human body through the mucosa such as the conjunctiva or disrupted skin [4]. Antibodies against *Leptospira* can be detected by ELISA three to seven days from the day of onset of symptoms, and somewhat later by agglutination testing, which optimally uses paired acute and convalescent samples [4, 5]. As serum antibodies increase, *Leptospira* disappears from circulation. However, antibodies may persist for a few months to several years [6].

Diagnostics for leptospirosis are interpreted according to the known natural history of leptospiral infection. Diagnostics are classified into four main categories: direct visualisation of bacteria through dark field microscopy; culture isolation; serological techniques to detect antibodies; and molecular techniques to detect nucleic acids of pathogenic *Leptospira* [7]. Direct visualisation, culture, molecular techniques, and antigen detection aim to diagnose during the leptospiraemic (acute) phase of the disease. In contrast, antibodies are aimed at the diagnosis during the immune phase. Despite the availability of different techniques, none provides higher validity than any individual test; some studies have shown higher accuracy when more than one test modality is combined [7]. Direct visualisation is limited in the sensitivity and specificity [8]. *Leptospira* culture has also shown very low sensitivity despite its high specificity. It is limited by the need for prolonged incubation and slow growth to provide clinically actionable results [9]. The pathogenic members of the genus *Leptospira* are highly diverse, comprised of multiple species and hundreds of serovars [9, 10]. Enzyme-linked immunosorbent assay (ELISA), lateral flow immune assay (LFIA), and the microscopic agglutination test (MAT) are some of the major serological tests used to detect antibodies [11–13].

A major limitation of serological techniques is that they cannot be used for diagnosis during the acute phase in most patients. Convalescent samples are optimal for serological diagnosis in terms of test performance [12]. Still, by the time of a second sample, the disease is cured, and the patient can be discharged from the hospital (or the patient has died). Antibody-based techniques have shown more than 80% sensitivity and specificity values; however, higher variability of validity was observed in different geographical locations [14, 15]. Molecular techniques are widely used in resource-available settings and adapted to resource-limited settings because of superior test performance. However, the sensitivity of PCR as an individual test is yet to be optimised due to its low sensitivity despite near 100% specificity [16]. Due to all these limitations, surveillance case definition has become the most common tool to diagnose leptospirosis in resource-poor settings. However, the surveillance case definition was validated compared to other diagnostic tests, with several limitations. Although surveillance case definition has shown sensitivity and specificity of over 70%, conclusions cannot be drawn due to the limitations of the standard test compared [17].

Limitations of diagnostic testing and reporting for leptospirosis suggest that confirmed cases underestimate the actual disease burden. Based on resource availability, clinical suspicion, and many other reasons, the tests performed are varied across the globe. However, MAT is still considered the standard test in many settings, while qPCR is increasingly used as a clinically relevant diagnostic method. Therefore, we hypothesised that the disease burden estimation depends heavily on the diagnostic tests, and the evaluation of underestimation for specific tests is required to understand the global disease burden better. This study carried out a new assessment of optimal samples and timing and type of tests to diagnose leptospirosis.

## Methods

### Ethical statement

Informed written consent was obtained from all patients before sample collection. The ethical clearance for this study was obtained from the Ethics Review Committee of the Faculty of Medicine and Allied Sciences, Rajarata University of Sri Lanka. (Protocol No. ERC/2015/18)

### Study design and setting

**Fig 1:**
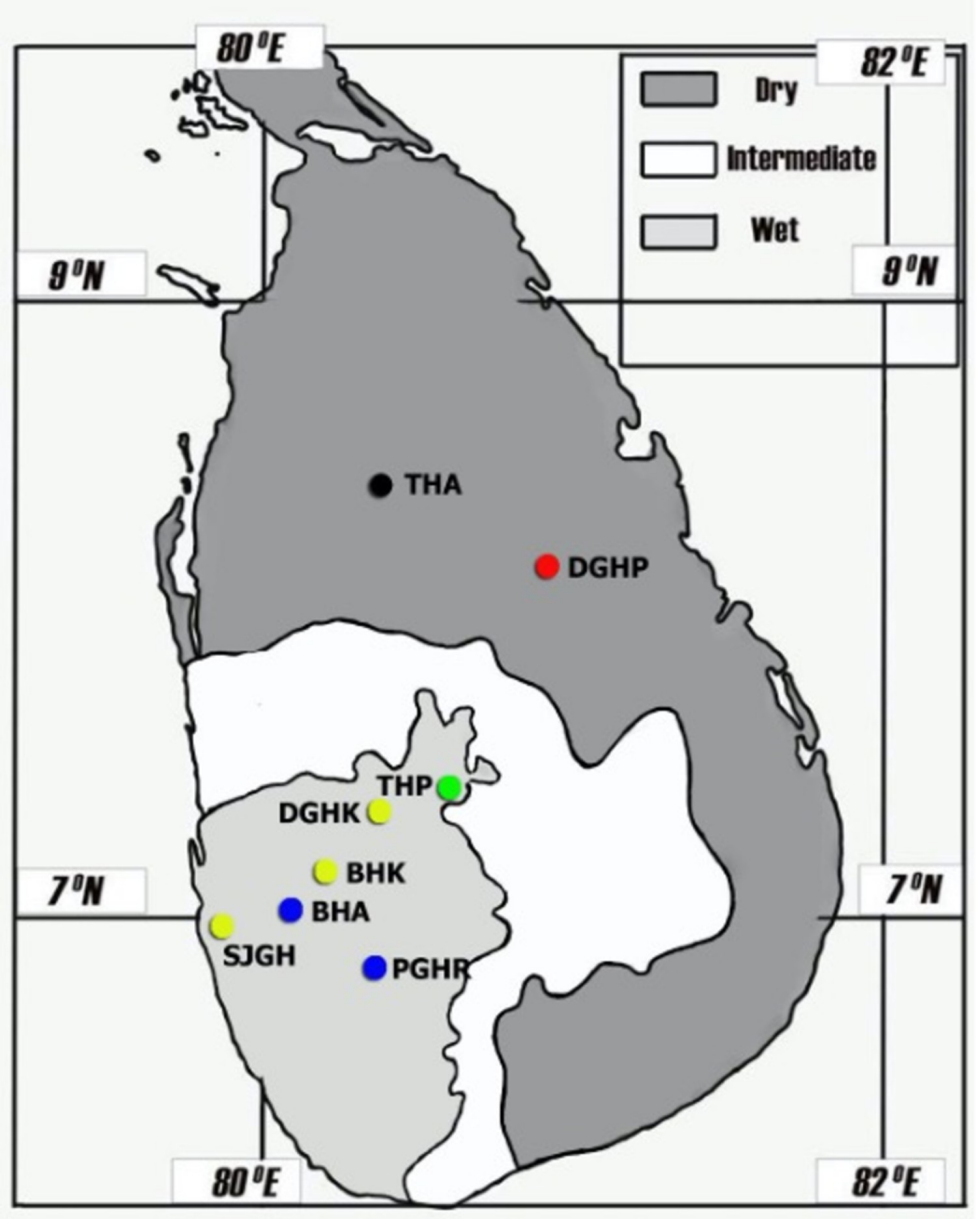
Distribution of study settings in relation to climate zones of Sri Lanka. Black: Blood, urine, and culture were collected from undifferentiated febrile patients throughout the period; Green: Blood, urine, and culture were collected from undifferentiated febrile patients during 2016, 2017; Red: Blood, urine, and culture were collected from probable leptospirosis patients during 2018, 2019; Blue: Blood, urine, and culture were collected from undifferentiated febrile patients during the flood season of 2017; Yellow: Only cultures were received.

This paper is a sub-study of a sizeable clinic-epidemiological study conducted in multiple hospital settings in Sri Lanka. The protocol of the parent study was published previously [17]. A hospital-based prospective study was carried out with recruitment at multiple Sri Lanka hospitals from 2016 to 2019 (Fig 1). The original protocol, isolation of *Leptospira*, and part of the microscopic agglutination test results are already published [9, 18]. The primary study sites were Teaching Hospital Anuradhapura (THA) and Teaching Hospital Peradeniya (THP). In addition, base Hospital Awissawella(BHA) and Provincial General Hospital Rathnapura (PGHR) were included during the flood season of the year 2017. Routine samples from probable leptospirosis patients were received for diagnosis from District General Hospital Polonnaruwa (DGHP). From District General Hospital Kegalle(DGHK), Base Hospital Karawanella (BHK), and Sri Jayawardanapura General Hospital (SJGH), culture samples were received from clinician-suspected typical cases of clinical leptospirosis for retrospective disease confirmation. These study sites represent high and low endemicity, dry and wet zones, and low and intermediate altitudes.

### Patients and recruitment

**Fig 2:**
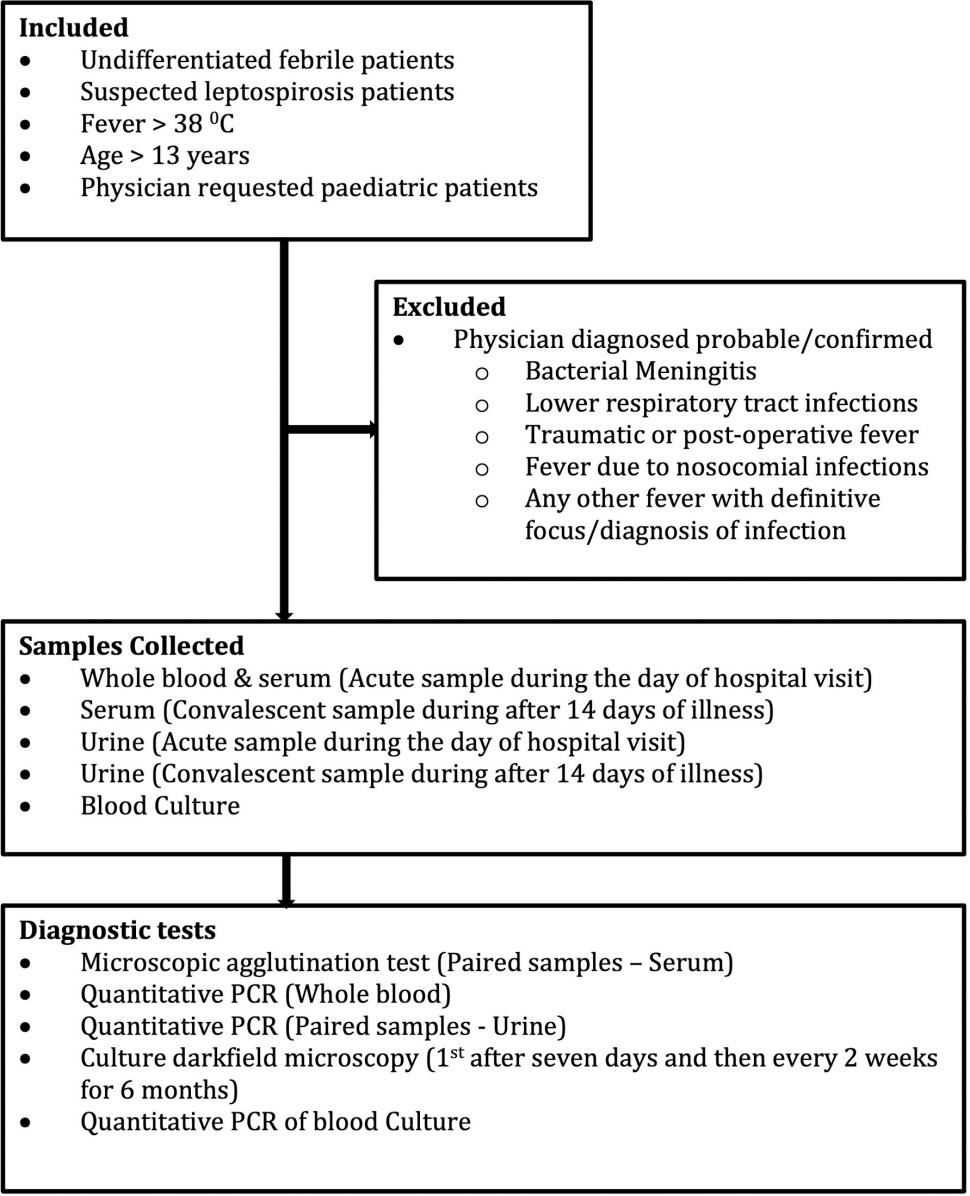
Study flow chart, inclusion-exclusion criteria, and diagnostic tests.

Fig 2 demonstrates the inclusion and exclusion criteria for the study. Samples from undifferentiated febrile patients were collected from 4 hospitals, and samples from clinically suspected leptospirosis were received from four other hospitals. Samples were collected from acute undifferentiated febrile (temperature> 38°C) patients from THA, THP, PGHR and BHA. In addition, samples from clinically suspected leptospirosis patients were received from DGHP, DGHK, BHK, SJGH.

Although patient recruitment was done from adult wards (age> 13 years), samples were also received from paediatric wards for diagnostic purposes. In addition, physician-suspected probable or definite acute bacterial meningitis or lower respiratory tract infections (e.g., consolidated lobar pneumonia), traumatic or post-operative fever per physician discretion, fever due to nosocomial infections, and any patient with the confirmed diagnosis of a cause for the fever were also excluded.

### Disease confirmation

The samples collected were whole blood, serum, urine, and whole blood inoculated in EMJH culture media. As reported previously, sample collection and testing procedures for culture and MAT tests were done using standard protocols [18]. MAT positivity was defined as seroconversion or a four-fold rise in MAT titre in paired samples or having a single titre≥1/400, using a broad group of antigen panel with 24 Leptospira strains. Whole blood and urine samples were used for molecular diagnosis. DNA was extracted from 200 μL of whole blood using QIAamp DNeasy Mini kit (Qiagen, Germany). qPCR was performed using the CFX96 real-time PCR detection system (Bio-Rad, USA). We used two sets of primer pairs targeting 16s rRNA (Forward-5’-GCGTAGGCGGACATGTAAGT-3’, Reverse-5’-AATCCCGTTCACTACCCACG-3’) and LipL32 (Forward-5’ TGG CTA TCT CCG TTG CAC TC 3’, Reverse-5’ CCC ATT TCA GCG ATT ACG GC 3’) genes. Further details on qPCR were published previously [18]. All qPCR were performed with a standard curve, and all samples were run in triplicates.

Acute and follow-up urine samples were tested for qPCR. In addition to these traditional biological samples, we performed qPCR on randomly selected microscopically negative blood cultures after six months of observation. This was done after observing that some cultures had initial growth but then either died off or were not detected.

All cultures were discarded after six months of observation for growth. We categorised the patients as confirmed, probable, reactive and possible leptospirosis, as shown in Table 1 [19]. Those who did not fall under any of the categories were categorized as negative patients.

**Table 1:** Case definitions for the recruitment of subjects and diagnosing the patients.

Purpose	Category	Definitions
Recruitment	Clinically suspected case	• Leptospirosis is included as the first differential diagnosis by the physician OR/AND • Patients who fulfil the criteria of surveillance case definition
	Undifferentiated febrile patient	Fever > 38 ^0^C without having any other definitive focus for fever or any other confirmed diagnosis
Diagnosis	Confirmed case	A patient with clinical signs and symptoms consistent with leptospirosis and any one of the following a. fourfold increase in Microscopic Agglutination Test) (MAT) titre in acute and convalescent serum samples b. MAT titre ≥1:400 in single or paired serum samples c. isolation of pathogenic *Leptospira* species from a normally sterile site d. pathogenic *Leptospira* species DNA detected by qPCR. (when samples are run in triplicate, at least two are amplified)
	Probable cases	qPCR A patient with clinical signs and symptoms consistent with leptospirosis and only a single well from triplicates shows amplification
		MAT A patient with clinical signs and symptoms consistent with leptospirosis and having a reactive MAT test, but not conforming to the diagnostic titres mentioned under confirmed cases are also categorized as probable (in general) or reactive (under MAT).
	Possible/ febrile cases	All other patients were included in this category. Since the inclusion criteria has fever, all these cases could be leptospirosis. Some of the patients are actually clinically identified as leptospirosis, but no laboratory tests to support the diagnosis.

Urine samples were processed within two hours of collection according to a two-step protocol published by Paula et al [20]. Each 10mL was centrifuged at 3000 rpm (1000 *g*) for five minutes. The supernatant from each sample was taken into four micro-centrifuge tubes (2 mL each), centrifuged at 15000 rpm for 10 minutes, and supernatants were discarded. All four sediments were pooled, aliquot into two micro-centrifuge tubes, and stored at -20°C until used for qPCR.

200 μL of processed urine and 200 μL whole blood were subjected to extraction of DNA using QIAamp DNeasy Mini kit (Qiagen, Germany) according to manufacturer’s instructions. For the microscopically negative blood cultures, 1 mL of sample was aliquoted into a 1.5 mL microcentrifuge tube. DNA extraction was done using Thermo Scientific GeneJet Genomic DNA Purification Kit according to the manufacturer’s instructions.

Sample types, sample timing, and the diagnostic tests were compared as factors with a possible effect on reported numbers and possibly underestimating the actual caseload presented to the hospitals. Because empiric clinical diagnosis is the primary way to diagnose leptospirosis used in many places worldwide because of resource limitations, such clinical suspicion was included in the analysis. However, the clinical diagnosis varies widely among clinicians; thus, we used the surveillance case definition to classify patients retrospectively [17]. If the clinician’s first differential diagnosis is leptospirosis and those who reported fever, headache, and myalgia/muscle pain with jaundice, conjunctival suffusion, oliguria, or anuria at the time of presentation (or reported as having those symptoms before coming to the hospital) and those who have an exposure history were defined as having "clinically suspected" leptospirosis for this study.

## Results

### Study samples

From April 2016 to January 2019, biological samples from 1,734 patients were tested. The number of patients tested in 2016, 2017, 2018, and 2019 was 383, 730, 584, and 37, respectively (Table 2). Of the 1,734 participants, 298 were females, and gender was not included in 178 samples as they were sent as requested by the treating physicians. Most patients were from THA (n = 1017). Only 92 patients were recruited from the outpatient department, and the rest were hospitalised patients. For 97 patients, only whole blood inoculated culture media samples were received. There were 1513 undifferentiated febrile patients and 207 clinically suspected leptospirosis patients in the total sample. In addition, we received 14 samples from paediatric patients as requests of treating physicians.

**Table 2:** Patient samples and leptospirosis confirmation by year and hospital from April 2016 to January 2019.

Hospital	2016		2017		2018		2019		*Total*
	N	Confirmed (%)	N	Confirmed (%)	N	Confirmed (%)	N	Confirmed (%)	
**THA**	284	59(20.8)	243	111(45.7)	483	216(44.7)	7	0	***1017***
**THP**	99	19(19.2)	163	81(19.2)					***262***
**BHA**			167	26(15.6)					***167***
**DGHP** ^**1**^					101	31(30.7)	30	5(16.7)	**131**
**PGHR**			77	21(27.3)					***77***
**BHK** ^**2**^			60	20(33.3)					***60***
**SJGH** ^**1**^ ^**,2**^			10	0(0.0)					***10***
**DGHK** ^**2**^			10	2(20.0)					***10***
***Total***	***383***	***78(20*.*4)***	***730***	***261(35*.*8)***	***584***	***247(42*.*3)***	***37***	***5(13*.*5)***	***1734***

^1^ Samples received for diagnostic purposes only

^2^ Only the cultures were received. **THA-**Teaching Hospital Anuradhapura, **THP-**Teaching Hospital Peradeniya, **BHA-**Base Hospital Awissawella, **DGHP-**District general hospital, Polonnaruwa, **PGHR-**Provincial general hospital Rathnapura, **BHK-**Base Hospital Karawanella, **SJGH-**Sri Jayawardanapura General Hospital, **DGHK-**District general Hospital Kegalle.

Through a panel of diagnostic tests and different samples, 591 (34.1%) patients were confirmed as having leptospirosis and 297 (17.1%) were categorised as probable, and another 846 (48.8%) as possible/febrile patients according to the definitions given in Table 1. A detailed description of the numbers positive by each method is displayed in Table 3.

### qPCR

qPCR was done on 1606 patients, of which 1,455 were whole blood samples. In addition, qPCR was performed for 630 acute and 195 convalescent urine samples. Three hundred fifty-three microscopy-negative *Leptospira* cultures were tested using qPCR at six months. Altogether, 455/1606 (28.3%) patients were tested positive, while another 286/1606 (17.8%) patients had one positive out of triplicates samples and were labelled as "probable" cases as defined in Table 1.

### MAT

MAT was performed on 1389 subjects, and of them, we received 302 follow-up samples. Of these, 153/1389 (11.0%) patients were confirmed as having leptospirosis. Of these 153 patients, 101 (66.0%) were diagnosed based on the acute sample, and an additional 52 (34%) patients were confirmed as leptospirosis after analysing the follow-up samples. Of the 302 follow up samples, 241 (79.8%) were labelled as non-reactive based on the results of the acute sample, and of them, 39/241 (16.2%) were confirmed as leptospirosis according to the results of the follow-up sample. (Table 3)

**Table 3:** Leptospirosis disease confirmation according to different sample types and tests among 1734 undifferentiated febrile patients and clinically suspected leptospirosis patients from Sri Lanka 2016–2019.

Method and sample type*	n	%
**qPCR**		
***Whole blood (N = 1455)***		
Confirmed	322	22.1
Probable	207	14.2
Not detected	926	63.6
***First Urine sample(N = 630)***		
Confirmed	67	10.6
Probable	65	10.3
Not detected	498	79.0
***Convalescent Urine(N = 195)***		
Confirmed	5	2.6
Probable	18	9.2
Not detected	172	88.2
***Culture qPCR(N = 353)***		
Confirmed	79	22.4
Probable	53	15.0
Not detected	221	62.6
**MAT**		
***Acute sample (N = 1389)***		
Positive	101	7.3
Reactive	124	8.9
Negative	1164	83.8
*Paired samples(N = 302)		
Positive	72	23.8
Reactive	47	15.6
Negative	183	60.6
**Culture *(N = 1192)***		
Positive	25	2.1
Negative	1167	97.9

*These categories are not mutually exclusive; hence, they may add up to >100%.

### Comparison of the diagnostic methods

We compared the qPCR of cultures with the qPCR for whole blood samples in the same patient. For 307 patients, both were done. Of them, 49 were positive for qPCR on cultures. Of these, 13 (26.5%) were detected through whole blood qPCR at the acute stage, another 5 (10.2%) were labelled as probable, and in 31 (63.3%), whole blood qPCR was negative. However, of the 101 cases confirmed through whole blood qPCR in this subsample, only 13 (12.9%) were positive in culture qPCR.

**Fig 3:**
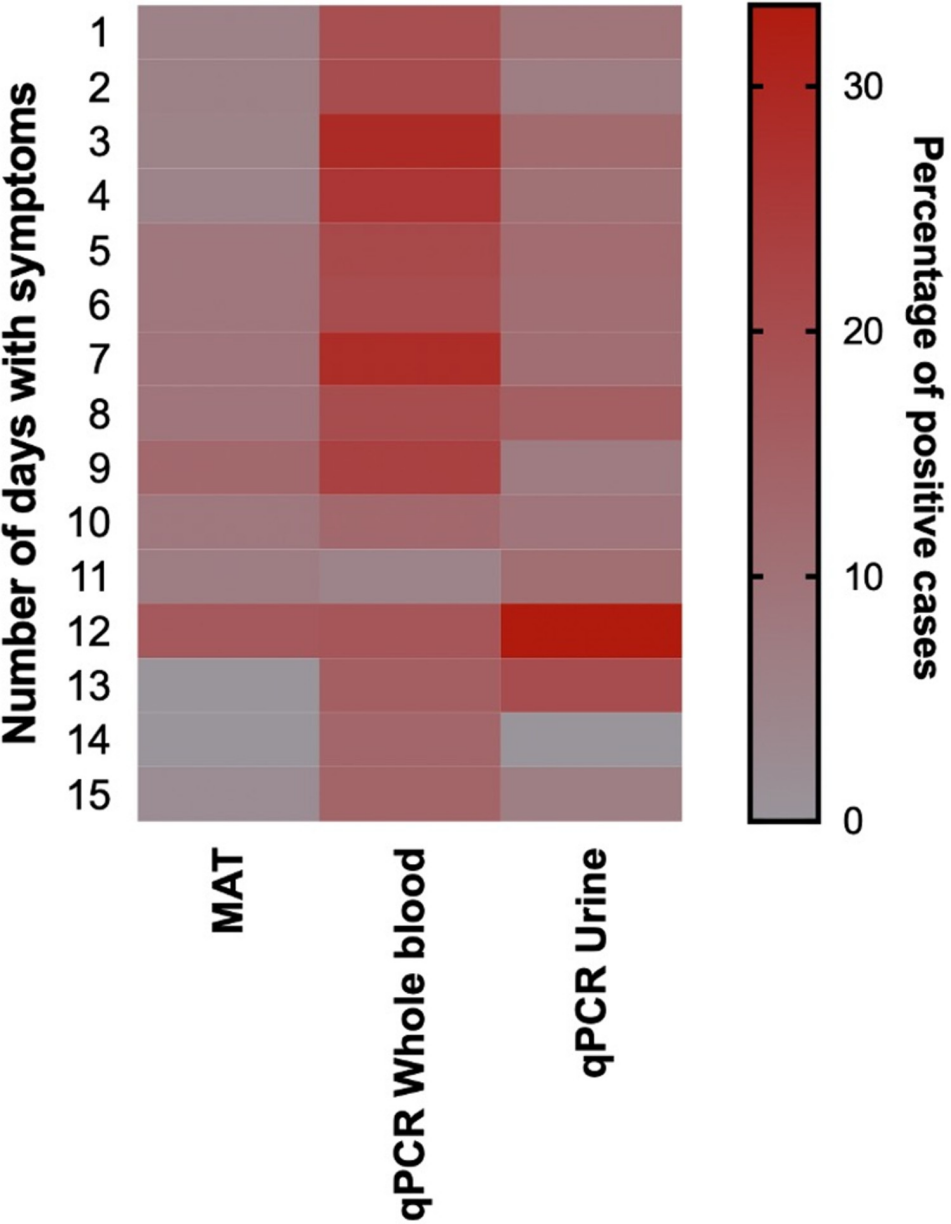
Heat map showing the percentage of positive cases by patient-reported day of illness, until day 15 of patient-reported symptoms.

The heat map includes only the acute samples, and therefore the results are displayed only for up to 15 days. (Fig 3). Patients admitted to the hospital after 15 days of illness were excluded from the Heat Map. High positivity of urine samples was seen on days 12 and 13. Sometimes MAT samples provided positive results on the first day of patient-reported illness, and the percentage positive increases after day 5. Urine qPCR and MAT results were not correlated. qPCR positivity is increased after day 12, while MAT positivity is reduced. However, only day 15 qPCR positivity is significantly higher than MAT (p = 0.004), while no statistically significant difference was observed between the positivity on days 12, 13 and 14. Further details regarding the heatmap including the 95% Confidence intervals are included in S1 Table.

### Demographic profile of confirmed cases

**Fig 4:**
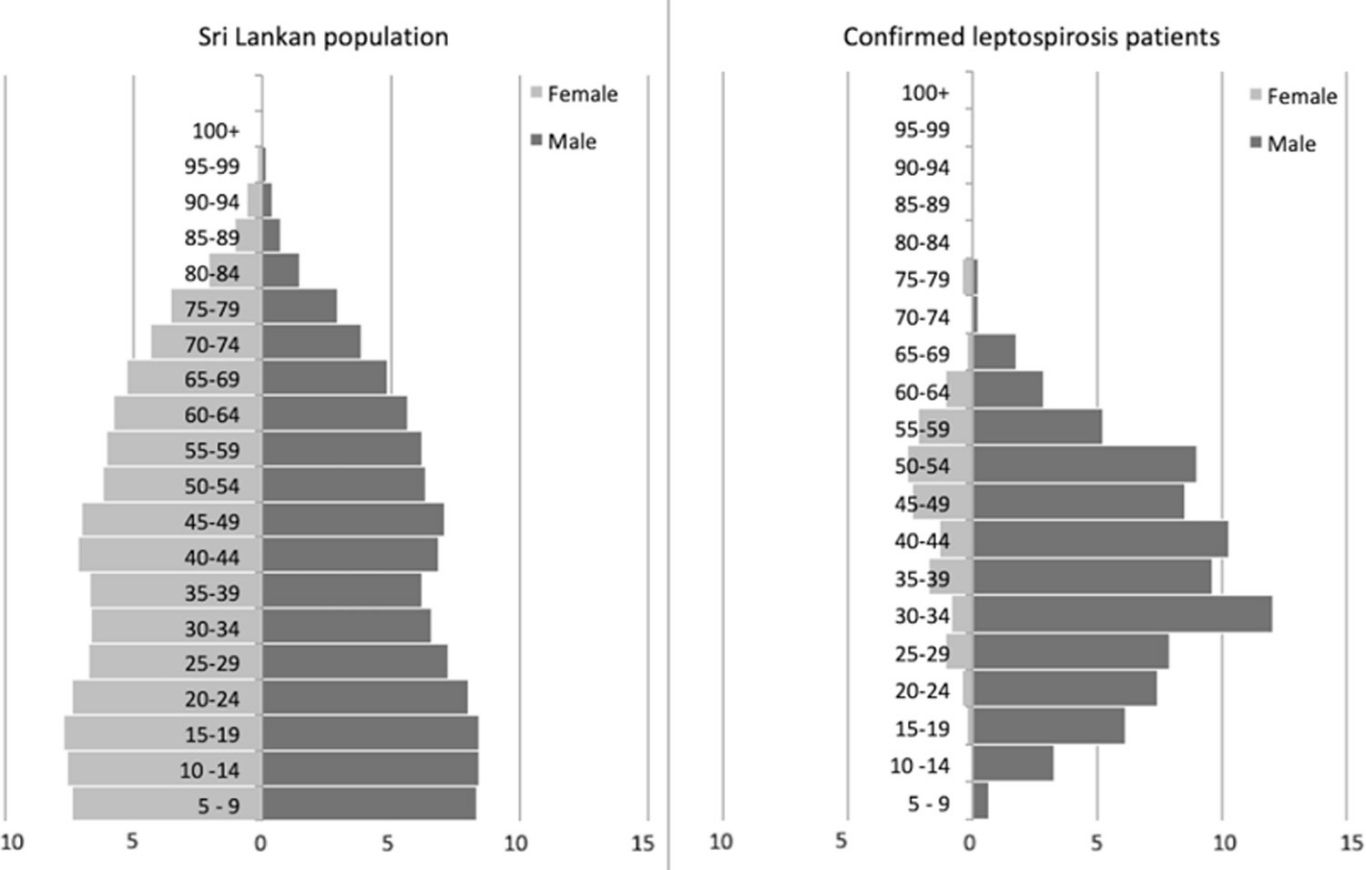
Comparison of the distribution of confirmed cases of leptospirosis with the general population age structure in Sri Lanka.

Of the 591 confirmed cases, demographic details were available for 514. Though 19.2% of the total sample were females, only 15.0% among confirmed cases included female patients (Chi-square 4.58, p .032). Comparing the demographic profile of the Sri Lankan population with confirmed leptospirosis cases, confirmed patients are predominantly males aged 20–60 age (Fig 4).

### Diagnostic method-based underestimation of leptospirosis

**Fig 5:**
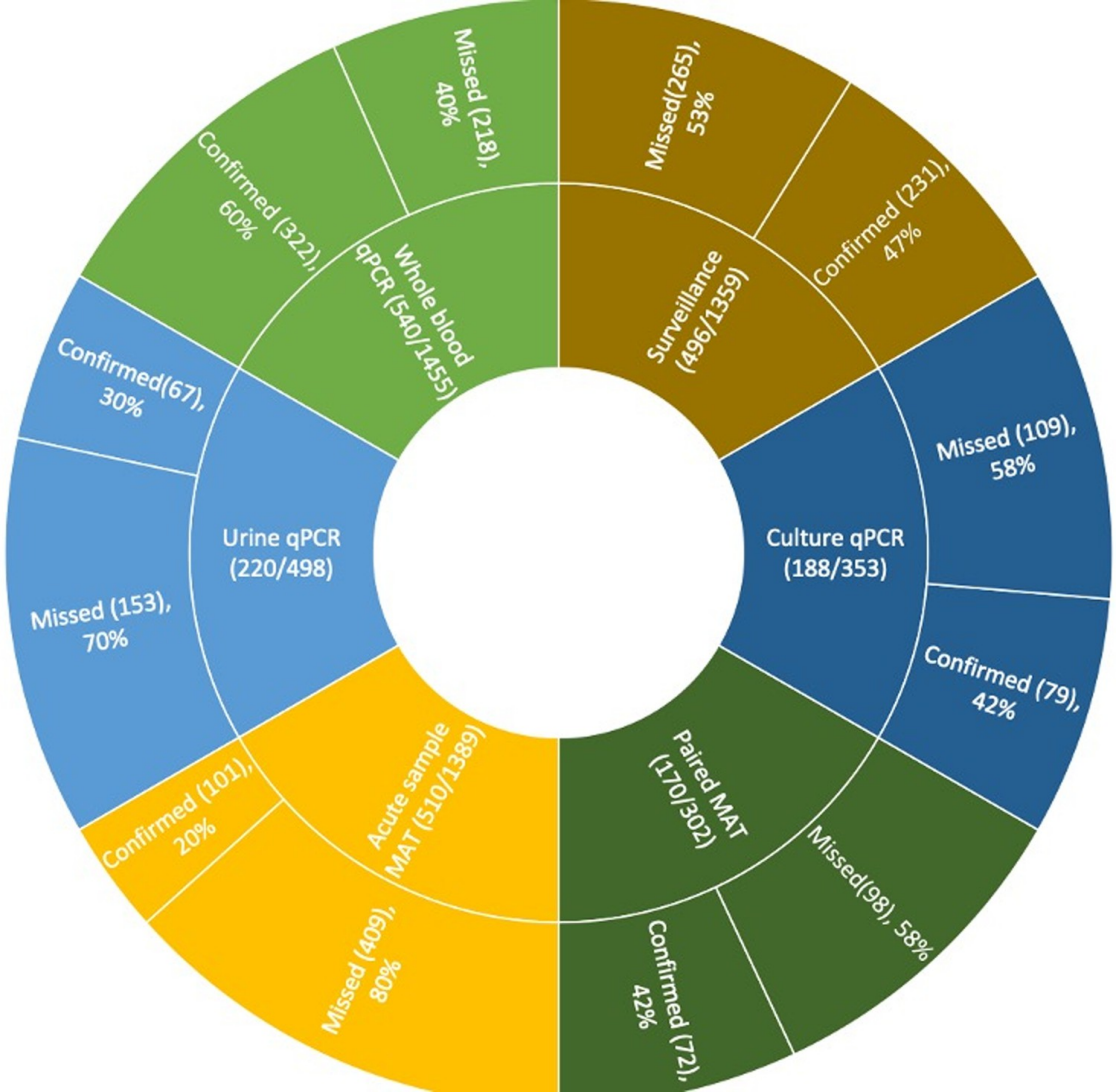
Diagnostic method-based underestimation of leptospirosis in clinical settings. The total number of tests done to calculate proportions is indicated in the inner circle. Figure legend: The inner circle shows the number of samples tested using each test (denominator) and the number of confirmed cases included in that sample (numerator). The outer circle indicates the percentage of confirmed cases detected or missed by each test (from the numerator of the inner circle). For example, 1455 samples were tested using whole blood qPCR. Of these 1455 patients, 540 were finally confirmed as leptospirosis. However, only 322 were tested positive using qPCR, and it missed approximately 40% of confirmed cases.

Whole blood qPCR, urine qPCR, acute sample MAT, and the surveillance case definition were compared to determine the diagnostic method-based underestimation of the caseload in clinical settings. In addition, paired-sample MAT and qPCR on culture samples were added to estimate the probable underestimation in epidemiological studies with long-term follow-up. Fig 5 shows the percentages of missing cases by each diagnostic method.

MAT provided the least accurate estimate based on the acute sample diagnosis, with inaccurately labelling 80% of confirmed positive samples. Urine qPCR also underestimated the cases, with 70% of tests missing the diagnosis. qPCR using whole blood provided the best results, missing only 40% of cases. However, for long-term follow-up with paired samples, both MAT and culture qPCR missed 58% of cases.

Because most confirmatory tests substantially underestimate leptospirosis disease burden, underestimating using surveillance case definitions was examined. Only 47% of all the confirmed cases presented were compatible with the surveillance case definition, still capturing more than MAT and urine qPCR. However, surveillance case definition suffered from a lack of specificity.

## Discussion

The most important finding of this study is the gross underestimation of hospitalised cases. Global disease burden estimates for leptospirosis are mainly based on country-level datasets [2, 3]. The country-level datasets are based on clinical diagnosis and reporting in tropical countries. Our analysis shows that the clinical diagnosis-based underestimation may be around 50% of actual hospitalised cases. Where the reporting is based on MAT, the underestimation could be as high as 80%. Based on the light of these findings, adjustments are required for the global disease burden estimates and the country level disease estimates with a 50–80% overall increase of actual hospitalised patients.

Despite significant ongoing advances in the field, a "gold standard" test to diagnose leptospirosis has yet to be established [17, 21, 22]. Based on lack of consensus, even the "standard test" is debated [22]. Issues related to the diagnosis of leptospirosis have led to a wide variation of practices across the globe, partially based on the resource availability to diagnose leptospirosis definitively. While the diagnosis directly affects the clinical management of the individual cases, it also affects the disease burden estimates, essential for informing public health policy [1].

Global disease burden estimates of leptospirosis have been done based on surveillance or hospital data [2] with important efforts to avoid bias. Here we show that in a highly endemic region for leptospirosis, underestimating actual hospitalised cases is as high as 80% when the most widely used "standard test," MAT, is used to diagnose acute febrile patients. The low sensitivity of MAT is well known for acute samples, having been estimated using clinical samples with varying degrees of uncertainty in terms of clinical metadata. Many clinical studies have an inherent selection bias in which inclusion criteria partially depend on typical leptospirosis features [1] suspected by the treating physician. When the patient recruitment is biased with "typical" symptoms, it is a proxy feature of the immune phase of leptospirosis and more likely to have a positive MAT test, thus showing a higher clinical validity [17]. Such bias is present even if samples are taken early in the illness [21]. In the present study, we have recruited patients based on fever alone and without any other diagnosis (undifferentiated febrile patients). This can lead to the identification of many patients who would not otherwise have been categorised as "clinical leptospirosis." This recruitment was facilitated by having a dedicated clinical data collector who had recruited patients independently without influence from the treating physician. This deliberate study design determined a very low sensitivity of MAT, even with paired samples. We propose that these estimates better represent the accurate picture of leptospirosis [21].

As previously demonstrated, qPCR shows the highest yield and clinical utility and will have the least effect on disease burden underestimates [23]. The present data shows that though qPCR may not be a "gold standard," it should be a standard test for confirming a clinical diagnosis of leptospirosis. qPCR should be possible across the globe now, given that most countries now have access to qPCR equipment and trained personnel due to the COVID-19 pandemic. It has been reported that there has been a rapid increase in PCR capacity over a year and is likely to continue to increase [24]. In this study, we reiterate that qPCR is an effective diagnostic modality throughout the first 15 days (Fig 5), contrasting to the traditional belief that the test has to be performed during the first few days of illness because the circulating *Leptospira* is rapidly cleared from the bloodstream by the immune phase of the illness [4]. However, the best results are seen in the first nine days of illness. Even with a comparatively higher detection rate, suboptimal sensitivity is still an issue related to qPCR, as observed previously [16]. Inability to capture the lower level of leptospiraemia could explain the low sensitivity of qPCR [25], and improving lower leptospiraemia requires further investigation.

Sample timing in this study confirms the generally known natural history of leptospiral infection. It is worth noting that whole blood qPCR is better than MAT even during the second week of illness, confirming our previous observations [23]. Therefore, our observations differed from the best timing for the testing introduced by Ooteman *et al*. [26]. One unexpected observation of this study is increasing qPCR positivity and reducing MAT positivity after day 12, although they are not statistically significant except on day 15. Combined with our clinical observations, this could be confounded by patients’ recollections of the timing of fever. For example, when patients come to the hospital on day 12 with fever, they may be more likely to connect two different illnesses and report it as a single condition. If they had any other tropical fever a week back, for example, and were admitted to the hospital this time due to fever, they will have positive qPCR while the MAT is still negative. This observation is confirmed by having more positives in the convalescent samples but not in a single sample that comes late. Therefore, the observed positivity after day 12 is a systematic "error" in this study that may be common to other leptospirosis studies in endemic tropical regions.

We have observed that 42% of microscopical-negative cultures as positive through qPCR. Culture isolation is considered time-consuming, cumbersome, and a meagre yield procedure [9]. We hypothesise that there may be some degree of growth in most cultures, but organisms died for many reasons—perhaps species/serovar-specific factors—requiring further exploration. Also, empirical antibiotic administration or antibiotic misuse before taking blood for culture can lead to a lower concentration of *Leptospira* in the samples. Studying the culture concentrations and optimal procedures will change the low sensitivity of cultures, a major challenge in the field of leptospirosis.

On the other hand, the qPCR of culture is much better than paired sample MAT for retrospective diagnostic purposes. In our sample, 63.3% of culture qPCR positive samples had a negative qPCR for whole blood. This finding will also be a game-changer for future research. If we can further study this, the patients are not required to return for the convalescent sample; instead, one can have a culture tube for a few weeks and test it again to improve the disease confirmation. How long a culture should be maintained before qPCR requires further studies. A major limitation of surveillance case definition is the lack of specificity. When the specificity of low, there is a higher probability of having false-positive results. Diseases such as dengue and hantavirus infection, which mimics leptospirosis, and epidemiological factors contributing to the conditions are similar; there is a higher probability that the surveillance case definition is positive for leptospirosis, although the true diagnosis could be something else [27–29].

Based on the findings reported here that are based on minimisation of clinician bias combined with different biological sample testing, we recommend the following: 1) whole blood qPCR should be the standard test for the diagnosis of leptospirosis for clinical purposes until day 10 of the reported disease; 2) MAT should be limited to only if the serological diagnosis has an epidemiological interest. If MAT is to be done, it should be after day 5 of illness; 3) current disease burden estimates of leptospirosis should be revised in the light of gross underestimation of disease due to issues in the diagnostic methods and bias in selecting patients for testing, and 4) qPCR testing of microscopically-negative cultures should be done before discarding to increase the yield in research setting without an additional patient visit.

## Supporting information

S1 Table MAT, Whole blood qPCR and Urine qPCR positive proportions with 95% Confidence Intervals from day one (1) to day fifteen (15) of the illness. (XLSX)
